# Using Intervention Mapping and Behavior Change Techniques to Develop a Health Promotion Intervention on Endocrine Disruptors: Development Study

**DOI:** 10.3390/ijerph22020216

**Published:** 2025-02-04

**Authors:** Camille Lassalle, Véronique Régnier, Laetitia Marcucci, Julien Masson

**Affiliations:** 1 Laboratoire Parcours Santé Systémique (P2S), Université Claude Bernard, Lyon 1, 69622 Villeurbanne Cedex, France; lassalleccm@gmail.com; 2 Institut PRESAGE (Institut Universitaire de Prévention et Santé Globale), Université Jean Monnet Saint-Étienne, 42270 Saint-Priest-en-Jarez, France; veronique.regnier@univ-st-etienne.fr (V.R.); laetitia.marcuccihilaire@gmail.com (L.M.); 3 Laboratoire Education Culture Politiques, Université Lumière, Lyon 2, 69365 Lyon Cedex, France

**Keywords:** endocrine disruptors, adolescents, health education, intervention mapping, COPE ADOS, public health, environmental risks

## Abstract

Endocrine disruptors (EDs) are substances that interfere with the endocrine system, posing risks to health across various life stages, particularly during adolescence when hormonal changes are pronounced. Despite the recognition of adolescents as vulnerable, there have been few interventions targeting their exposure to EDs. This study developed the COPE ADOS program using the intervention mapping (IM) framework to enhance adolescents’ knowledge and skills in identifying and mitigating exposure to EDs. The IM framework guided the creation of the program through four steps: conducting a needs assessment, formulating program objectives, selecting relevant behavioral theories, and developing a logical model. The need assessment conducted through focus groups revealed significant knowledge gaps and misconceptions about EDs among adolescents, leading to the establishment of six performance objectives aimed at addressing attitude, knowledge, risk perception, self-efficacy, and skills. As a result, 15 educational tools were created. The COPE ADOS program represents a novel, collaborative effort tailored to the needs of students and demonstrates the potential of the IM framework in developing effective health interventions for adolescents. Future research should evaluate the impact of this program on reducing ED exposure among high school students.

## 1. Introduction

The term endocrine-disrupting (ED), introduced in the early 1990s, refers to an external substance or combination that alters the functioning of the endocrine system, leading to adverse health effects in a complete organism, its progeny, or specific populations, as defined by Damstra et al. in 2002 [1].

Research indicates that endocrine disruptors manifest a spectrum of health effects. The *State of the Science* report on these chemicals outlines their impact on female and male reproductive health, sex ratio imbalances, thyroid-related disorders, hormonal-related cancers, neurodevelopmental disorders in children, adrenal disorders in humans, metabolic disorders, bone disorders, immune function, and diseases in humans [2].

Exposure to endocrine disruptors during specific life stages can heighten susceptibility. Early childhood and pregnancy, recognized as vulnerable periods, have prompted numerous interventions to address and prevent exposures during these critical times [3]. The adolescence phase, marked by pubertal sexual activation, emerges as a crucial period, emphasizing the need to examine and mitigate the impact of endocrine-disrupting substances during this developmental stage [3].

Despite this, there has been a notable lack of targeted initiatives addressing endocrine disruptor exposure in the adolescent population. This issue is particularly pronounced in the French context, where interventions for pregnant women and newborns exist, yet insufficient efforts have been made for adolescents. However, initiatives like awareness workshops initiated in 2019 aim to decrease endocrine disruptor exposure among pregnant women [4]. However, comprehensive actions for adolescents have been limited. One noteworthy exception is a commitment made by a French region to raise awareness among high school students: starting in 2021, adolescents participated in a scientific experiment to evaluate their exposure to nine phthalates. Furthermore, the Environmental Health Network and the Ile-de-France Region, in collaboration with the IRES-Kudzu Science laboratory, conducted an extensive awareness project on endocrine disruptors. This project involved 10,000 high school students and included a study measuring the exposure of 155 students to 24 endocrine-disrupting substances. Notably, this was the first study of its scale to use the silicone bracelet method [5].

The necessity for targeted education within schools is underscored by the French government’s 2021 *Second Strategy on Endocrine Disruptors*. This national strategy aims to enhance self-protection and reduce environmental contamination from endocrine disruptors. The regulatory framework, primarily formulated at the European level, seeks to achieve a collectively satisfactory level of protection for both the population and the environment. One of its key actions, Action 7, focuses on raising awareness among students through educational initiatives promoting health in the school environment and through pedagogical activities [6].

Adolescence is a critical period of sensitivity to endocrine disruptors, yet existing educational tools and programs have largely overlooked this vulnerable stage. To address this significant gap, the COPE ADOS program was created. COPE ADOS, an acronym for Connaissances et Représentation des Perturbateurs Endocriniens chez les Adolescents (translated as Knowledge and Perceptions of Endocrine Disruptors Among Adolescents), is a novel initiative dedicated to equipping adolescents with the knowledge and practical skills needed to identify and protect themselves from endocrine disruptors. The program employs co-creation methods specifically tailored to adolescents, using a bottom-up approach informed by long-standing evidence from health behavioral theories [7]. Its primary aim is to create and evaluate an educational framework that enhances adolescents’ understanding of endocrine disruptors and their ability to adopt protective behaviors.

To achieve this goal, a co-creation team comprising professors, school nurses, librarians responsible for creating educational tools within their institutions, and researchers collaborated with students. Their collective efforts were directed at creating relevant and targeted tools to educate and empower adolescents regarding their exposure to endocrine disruptors. The study consisted of three steps: (1) a preliminary qualitative study to understand adolescents’ knowledge and perceptions of endocrine disruptors [8]; (2) the development of educational tools; and (3) the testing of these tools among high school students in the Auvergne Rhône Alpes region in France.

This paper aims to delineate the steps involved in creating these diverse educational tools.

## 2. Materials and Methods

This research paper presents a comprehensive application of the intervention mapping (IM) framework [9] to develop and adapt an evidence-based health education program, COPE ADOS. COPE ADOS is designed to enhance the knowledge and skills of adolescents in identifying and safeguarding against endocrine disruptors.

Intervention mapping (IM) is a method for constructing health interventions. IM involves co-constructing the intervention with stakeholders, using scientific data, and selecting psychosocial behavior theories. IM consists of 6 steps to guide researchers [9].

1. Conduct a needs assessment;
2. Formulate program objectives and select associated determinants;
3. Choose appropriate theories in social and human sciences and intervention methods;
4. Develop the program’s logical model;
5. Plan the adoption and implementation of the program;
6. Plan its evaluation.

The program was developed using the first four steps of the IM: (1) conduct a needs assessment; (2) formulate program objectives and select associated determinants; (3) choose appropriate theories in social and human sciences and intervention methods; and (4) develop the program’s logical model. The originally planned steps 5 and 6 of the mapping implementation program were not carried out in the context of this study because the adoption and implementation were governed by the rules imposed by the French national education system and the teaching staff.

### 2.1. Step 1: Conduct a Needs Assessment and Define the Logical Model of the Problem

In the first step of the methodology, the public health issue is analyzed, followed by a study of risky behaviors and their determinants [9].

The first step of the IM (conducting a needs assessment) was carried out in 2021 and 2022 using focus groups to gather adolescents’ perspectives on EDs [8]. Details on the need assessment framework are available in Appendix A.

Additionally, the IM involves using theories in the social and human sciences to build the solution model. Findings from the focus groups were therefore linked to social theories to provide a theoretical background that would support the program’s development. This involved constructing a program that incorporated the concepts highlighted in the findings, which were emphasized by the target population.

### 2.2. Step 2: Formulate Program Objectives and Select Associated Determinants

According to the IM methodology, there are three categories of objectives:

1. Distal objectives are the expected effects of the program on health status and/or quality of life;
2. Performance objectives are the expected effects of the program on behaviors in the target population;
3. Change objectives are the expected effects of the program on determinants of behaviors.

Following the IM methodology, program objectives were elaborated.

The distal objective of the program was determined by the research team at the project’s initiation. This objective was subdivided into performance objectives, also called educational objectives. These objectives were the expected sub-behaviors to be achieved by the target group to achieve the program’s main objective [9]. By cross-referencing determinants with performance objectives, more specific change objectives were translated from more general performance objectives. 

The performance (educational) objectives were determined in partnership with the co-construction team following the analysis of focus groups. By aligning the performance objectives with determinants, change objectives were formulated by the researchers and validated by the co-construction team.

### 2.3. Steps 3 and 4: Choose Appropriate Theories in Social and Human Sciences and Intervention Methods and Develop the Program’s Logical Model

The third phase of the IM process entails the identification and selection of theoretical methods that are believed to influence changes for the determinants associated with each educational objective.

These steps were executed through two distinct approaches:

1. The co-creation team worked to identify and address the technical requirements necessary to implement the program effectively within the French educational context. This process included ensuring that the proposed tools were compatible with materials commonly available in schools, such as basic classroom equipment and technology. Additionally, the team took into account the standard duration of a course in France, which is typically 50 min, to ensure that the educational activities could be delivered within a single class session. Another critical consideration was to ensure that teachers could easily use the platform and implement the program autonomously without requiring direct support from the research team. This autonomy was a key recommendation from the co-creation team to allow educators to seamlessly integrate the program into their existing curricula. Following these requirements, the co-creation team suggested tools based on templates they had previously employed.
2. The second approach was carried out by the researchers. Starting with the change objectives and utilizing the compilation of theoretical methods provided in the intervention mapping guide [10], theories and tools for addressing the objectives were identified and subsequently validated by the co-construction team.

## 3. Results

The following sections summarize the outputs and findings that were produced using steps 1 to 4 of the IM methodology, which contributed to the development of the COPEADOS program.

### 3.1. Step 1: Conduct a Needs Assessment and Define the Logical Model of the Problem

The focus groups assessed adolescents’ knowledge and perceptions regarding endocrine disruptors (EDs). A total of 275 participants took part in the focus groups, with 155 students enrolled in the general education track and 118 in the vocational track. The median age of these participants was 16 years. Regarding gender distribution, 50.7% of the students identified as female, 48.5% as male, 0.4% as non-binary, and 0.4% chose not to disclose their gender. Within these focus group sessions, adolescents engaged in discussions focused on four primary themes: (1) their understanding and perspectives on endocrine disruptors; (2) how they perceived the associated risks; (3) the sources of information and research methods they relied on; and (4) their overall attitudes toward preventive measures. You can access more details on the focus group methodology in Appendix A.

The analysis of the focus group discussions unveiled specific needs within each of the discussed themes. To begin, there was a clear necessity to augment knowledge about endocrine disruptors. Notably, the participants exhibited limited knowledge about these molecules, their mechanisms of action, potential consequences, and existing regulations.

Furthermore, the data on perceptions of EDs suggested that the COVID-19 pandemic had influenced these perceptions. Participants recalled controversies, including references to nuclear power plants. Additionally, the focus groups identified a degree of confusion regarding bodily systems, such as the immune, circulatory, and digestive systems. Regarding the perception of risks, the analysis of the focus groups recommended the development of strategies aimed at providing adolescents with easily understandable protective measures. Moreover, the findings underscored the importance of considering the use of new information and communication technologies (ICTs), especially social media, to engage adolescents. Lastly, concerning the general attitudes of students towards their health, it became apparent that adolescents need to regain a sense of agency, particularly in managing their health, to avoid a sense of fatalism and guilt about their behaviors. 

Following the completion of the analysis, the concepts identified during the focus groups were integrated with different behavioral science theories to construct a logical model of the problem. This involved aligning the recognized needs of the focus groups with the theory of reasoned action, the theory of planned behavior, and Bandura’s self-efficacy theory. The COPEADOS project targeted behavioral determinants such as attitude, knowledge, risk perception, self-efficacy, and skills (Table 1) [11,12,13]. These determinants served as the foundation for developing and evaluating an educational program aimed at enhancing adolescents’ knowledge and abilities in identifying and safeguarding themselves against endocrine disruptors. For more detailed information on these theories, please refer to Appendix B.

The program concentrates on individual-level determinants, aligning with the preferences expressed by the co-creation team. The program’s direction was clarified to emphasize individual behavioral outcomes, aligning with the COPEADOS program’s objective of collaboratively constructing and evaluating an educational program that enables adolescents to better protect themselves from exposure to endocrine disruptors.

### 3.2. Step 2: Formulate Program Objectives and Select Associated Determinants

The objective, formulated as “creating and evaluating an educational program to improve adolescents’ knowledge and abilities in identifying and protecting themselves from endocrine disruptors”, was initially defined collaboratively by the co-creation team at the project’s commencement. This broad goal was subsequently dissected into six distinct performance objectives, as identified by the co-creation team and termed as educational objectives by the researchers engaged in the COPEADOS project. These performance objectives included:

1. Increase knowledge levels about EDs;
2. Increase the sense of control over environmental risks;
3. Be able to assess risks related to EDs in everyday life and/or professional situations;
4. Be able to implement appropriate prevention strategies;
5. Know how to identify reliable sources of information on EDs;
6. Reduce anxiety related to EDs and their exposure.

By aligning the performance objectives with the determinants derived from the social theories described in Step 1, change objectives were formulated by the researchers and validated by the co-construction team. Therefore, the six performance objectives were aligned with attitude, knowledge, risk perceptions, self-efficacy, and skills. 

To provide an example, the objectives of change for the initial performance goal of the program, “Increase knowledge levels about EDs” can be found in Table 2. A similar approach was employed to identify the objectives of change corresponding to each of the performance objectives.

### 3.3. Steps 3 and 4: Choose Appropriate Theories in Social and Human Sciences and Intervention Methods and Develop the Program’s Logical Model

To progress with the development of the program’s logical model, steps 3 and 4 required a series of 13 meetings with the co-creation team to achieve a consensus on the model. In the initial strategy, the co-creation team established a framework for the program, outlining three sessions, each with a unique objective. Session 1 was dedicated to evaluating students’ knowledge and perceptions. The co-creation team emphasized the need for tools in this session to enable a comprehensive assessment of students’ specific needs, allowing for the customization of the program accordingly. Session 2 focused on fostering the acquisition of knowledge and skills, while Session 3 was designed for consolidating contributions and facilitating a debriefing process.

Requirements from the co-creation team also stressed that each session should be limited to a duration of 50 min to align with the typical pace of high school activities. The program should be adaptable for use in both whole classes and small groups. The tools were to be provided by high school staff members who had already tested them, ensuring that facilitators at the secondary school could implement the program independently and without additional support. Each tool needed to be easily downloadable from a shared platform and accessible to facilitators responsible for student training. Clear technical guidelines were to accompany each tool, allowing facilitators to use them confidently without researcher assistance, fostering autonomy in delivering the program effectively.

Taking these criteria into consideration, a total of 15 tools were developed for the COPEADOS program, as detailed in Table 3.

Finally, researchers have developed frameworks that enable high school staff members to choose tools based on the number of sessions, allowing them to comprehensively address all the objectives targeted by the program (see Table 3). 

The implementation of the COPEADOS program targeted adolescents enrolled in secondary education programs across the Rhône-Alpes region of France. Recruitment included 20 high schools, evenly divided between general education (10 schools) and vocational education (10 schools). The study was conducted throughout 2023 and 2024, and the results will be presented and developed in a subsequent article.

## 4. Discussion

This paper does not assess the effectiveness of the intervention; this aspect will be addressed in a separate study. The ensuing discussion aims to explore various facets of the COPEADOS program’s development, highlighting both its strengths and weaknesses, and offering reflections on the experience.

The COPEADOS program represents a pioneering effort in health promotion, aiming to tackle the complex issue of endocrine disruptor exposure among adolescents.

By adopting intervention mapping as a systematic approach, our study crafted a theory- and evidence-based school-based program tailored to address the health and educational needs of adolescents. IM, elucidated by Bartholomew, Parcel, and Kok [9], furnished a structured framework encompassing needs assessment, determinant identification, and program design. Furthermore, insights from Kok et al. [14] provided a taxonomy of behavior change methods within the IM approach, guiding the selection of intervention strategies in our program.

Our approach to developing the intervention involved forming collaborative partnerships, where we actively incorporated the methodologies and insights as described by Lloyd et al. and Noel et al. [15,16]. Our engagement with school staff, community stakeholders, and researchers facilitated the co-creation of the program, ensuring its relevance and feasibility within the school environment. Leveraging community–academic partnerships empowered us to harness local expertise and resources to address the specific needs of adolescents. Tailoring interventions to specific environments and populations is essential for efficacy, informed by the ecological perspective advocated by Allen and Mohatt [17]. This approach enabled us to navigate constraints within the school setting, ensuring the program’s feasibility and sustainability. Our program development process aligned with contemporary theories of co-creation in public health, underscoring the importance of tailoring interventions to meet the evolving needs of diverse populations, as explored by Messiha et al. [18].

The utilization of the intervention mapping methodology uncovered both strengths and weaknesses in the formulation of the COPEADOS program. While furnishing a systematic framework for development, it also presented challenges, including time constraints and limitations in accommodating external regulations. Overcoming these challenges necessitated effective communication and the application of the socio-ecological approach, augmenting the precision and efficacy of need assessment conducted by the researchers and the school staff team. The inclusion of diverse stakeholders exemplifies a robust approach to customizing the program, ensuring its alignment with the school environment and the diverse needs of the target population.

The COPEADOS program introduces an innovative approach to addressing the intricate issue of endocrine disruptors among adolescents, serving as a pioneering initiative in health promotion. Rooted in the principles of intervention mapping (IM), this program diverges from conventional health promotion strategies by prioritizing empowerment and customization, both for students and school staff. Central to its design is the recognition of the various challenges and needs faced by adolescents concerning endocrine disruptors, emphasizing the importance of tailoring interventions to meet these specific requirements, whether in general education or vocational training, such as in the field of hairdressing.

A fundamental aspect of the COPEADOS program is the autonomy granted to school staff, distinguishing it from traditional health promotion strategies. This empowerment enables school staff to tailor interventions to the unique context and dynamics of their students, fostering greater alignment with their needs and concerns. Research by Morales-Garzón et al. [19] underscores the significance of community participation and co-creation in public health interventions, highlighting the value of empowering stakeholders to actively engage in program development. By cultivating a collaborative environment where educators have the agency to customize interventions, COPEADOS fosters a sense of ownership and engagement among school staff, thereby enhancing the program’s feasibility, sustainability, and impact.

The COPEADOS program embraces a multidisciplinary approach, with a particular emphasis on behavioral sciences, to navigate the complexities of adolescent development. By integrating insights from behavioral sciences, COPEADOS aims to equip adolescents with the requisite skills and self-efficacy to navigate the challenges posed by endocrine disruptors. Research by Kok et al. [14] furnishes a taxonomy of behavior change methods, offering a comprehensive framework for understanding the mechanisms underlying behavior change. This holistic perspective recognizes that behavior change is influenced by multifaceted factors beyond mere awareness, emphasizing the importance of addressing psychological, social, and environmental determinants of health behavior. Through its focus on behavioral sciences, COPEADOS endeavors to empower adolescents to make informed decisions regarding their health, thereby promoting positive and sustainable behavior change and mitigating the risk of exposure to endocrine disruptors.

In contexts where regulations may be slow to adapt or insufficient in providing protection [20,21], our study underscores the importance of empowerment programs. These initiatives empower individuals and communities to proactively manage their health, bridging the gap left by regulatory constraints. For instance, proposed legislation in France sought to address risks associated with per- and polyfluoroalkyl substances, though its effects would not be realized until 2027 [22]. This delayed timeline underscores the imperative for immediate action to address pressing health concerns. Empowerment programs play a pivotal role in equipping adolescents with the knowledge and skills to mitigate their exposure to harmful substances. By empowering individuals to take control of their health, these programs complement regulatory efforts and foster collective action toward broader systemic change, serving as vital catalysts for both individual empowerment and advocacy for timely regulatory reforms.

Our study acknowledges the limitations of a sole behavioral approach and aligns with the IM methodology, advocating for the incorporation of contextual and environmental factors. This holistic perspective critiques the exclusive focus on behavior and advocates for a more comprehensive understanding of health promotion interventions. To this end, we propose the development of more holistic programs that encompass not only individual behavior change but also broader socio-environmental determinants of health.

Such holistic programs could adopt a multifaceted approach integrating elements of education, community engagement, policy advocacy, and environmental modifications. For instance, in addition to providing adolescents with knowledge and skills to make healthier choices, these programs could advocate for changes in school policies to promote healthier environments, with the involvement of elected high school students on the Board of Directors within the framework of the CESCE (Committee for Health, Citizenship, and Environmental Education). This may include initiatives to enhance access to nutritious food options in school cafeterias, augment opportunities for physical activity during school hours, and foster supportive social norms around health behaviors.

Furthermore, these holistic programs could engage with the broader community to address systemic issues contributing to health disparities. Such programs should go beyond focusing solely on individuals and prioritize systemic actions, particularly in tackling endocrine disruptors. By addressing social determinants of health, including equitable access to healthcare and environmental health concerns, these initiatives can mitigate the disproportionate exposure of populations with lower socioeconomic status to environmental risks.

Examples of programs that could be merged to behavioral initiatives are highlighted on the CEREMA platform dedicated to environmental health [23]. These include town halls participating in the Healthy School Program, which promotes the selection of non-toxic school supplies and the adoption of Healthy Schoolbag campaigns free of endocrine disruptors and CMR (carcinogenic, mutagenic, and reprotoxic) substances [24]. Other actions involve the use of Eco-cert-certified products for building maintenance and the introduction of steam cleaning methods in secondary education institutions by the Nouvelle-Aquitaine region to ensure ecological and effective maintenance practices [25].

Additionally, awareness campaigns targeting vulnerable populations have been implemented in municipalities like Aubervilliers, where social precariousness is high, and 44% of the population lived in poverty as of 2014. With 66% of households including children, these campaigns aim to educate families about exposure to environmental pollutants, providing a vital resource for those facing elevated risks [26].

The call for more holistic programs underscores the significance of addressing the underlying determinants of health in conjunction with individual behaviors. By adopting a comprehensive approach that considers the broader social, economic, and environmental contexts in which health behaviors occur, these programs have the potential to effect lasting change and improve health outcomes for adolescents and communities alike.

## 5. Conclusions

This paper outlines the creation of the COPEADOS program, employing the intervention mapping (IM) methodology to enhance adolescents’ knowledge and awareness of endocrine disruptors. Notably, it represents the inaugural prevention program co-developed with school staff, specifically customized to meet the unique needs of students. The comprehensive process facilitated the collaboration of a diverse team, integrating theory and evidence to construct an effective intervention. The findings suggest that the IM protocol serves as a valuable guide for institutions seeking to implement tailored interventions for adolescents regarding endocrine disruptors. Future endeavors should focus on evaluating whether the implementation of such interventions in high schools can indeed result in significant reductions in endocrine disruptor exposure.

## Figures and Tables

**Table 1:** Theories, focus, and determinants result from the analysis of focus groups to create the logical model of change [11,12,13].

Theories	Focus	Determinants
Theory of reasoned action [11]	The theory suggests that contextual factors play a role in initiating behavioral change. Insufficient knowledge can hinder the adoption of new behavior, while attitude, influenced by beliefs, reflects positive or negative evaluations of behavior performance.	Knowledge attitude
Theory of planned behavior [12]	Perceived control can alter not only behavioral intention but also the behavior itself. Self-efficacy pertains to people’s confidence in their ability to take action that influences events in their lives. The belief in efficacy suggests that if individuals do not believe they can achieve the desired outcomes through their actions, they have little motivation to act or persist in the face of challenges.	Risk perception self-efficacy
Cognitive theory [13]	According to this theory, promoting change requires offering learning opportunities and cultivating skills for new behaviors and problem-solving.	Skills

**Table 2:** Change objectives aimed at increasing students’ knowledge levels about EDs.

Change Objective 1: Increase knowledge levels about EDs	**Attitude**	**Knowledge**	**Risk Perception**	**Self-Efficacy**	**Skills**
	The student expresses a positive feeling about benefiting from a prevention program on endocrine disruptors.	The student defines an endocrine disruptor.	The student recognizes the risks associated with endocrine disruptors.	The student expresses confidence in their knowledge of endocrine disruptors.	
					The student demonstrates an ability to identify endocrine disruptors.
	The student expresses a desire to take action on their exposure to endocrine disruptors.	The student mentions the specificities of endocrine disruptors.		The student believes that they have knowledge about endocrine disruptors	
		The student can recognize problematic substances.		The student is confident that they can take action on their exposure to endocrine disruptors.	

**Table 3:** List of tools created with the program objectives.

Tool Proposed	Targeted Program Objective	Description
Session 1: Collection of representation and concepts		
Affirmation and controversy		50 min Exchanges about proposed statements to provoke reflection.
Drawings		50 min Drawing proposals to elicit reflections.
Questionnaire		50 min Closed-ended question and answer session.
Photo expression		50 min By selecting an image, the student expresses their interpretation regarding endocrine disruptors.
Session 2: Knowledge contribution		
Informative videos	1. Increase knowledge levels about EDs. 3. Be able to assess risks related to EDs in everyday life and/or professional situations. 4. Be able to implement appropriate prevention strategies.	50 min After watching a short informative video, the student answers a knowledge questionnaire.
Game of 7 families	1. Increase knowledge levels about EDs. 3. Be able to assess risks related to EDs in everyday life and/or professional situations.	50 min While playing cards, the student encounters the names of different families of endocrine disruptors, their objects and locations, as well as general information about endocrine disruptors.
Posters *	1. Increase knowledge levels about EDs. 3. Be able to assess risks related to EDs in everyday life and/or professional situations.	Throughout the prevention program, informative posters will be available.
Article research and presentation	2. Increase the sense of control over environmental risks. 5. Know how to identify reliable sources of information on EDs.	50 min With a pre-selection of articles, students choose articles related to the designated theme.
Emotional weather forecast and self-efficacy guide	2. Increase the sense of control over environmental risks. 5. Know how to identify reliable sources of information on EDs.	50 min After sharing their emotions about endocrine disruptors, students set achievable goals to initiate behavior changes to limit their exposure to endocrine disruptors.
Session 3: Consolidation and debriefing		
Posters *	1. Increase knowledge levels about EDs. 3. Be able to assess risks related to EDs in everyday life and/or professional situations.	Throughout the prevention program, informative posters will be available.
Home-made deodorant	1. Increase knowledge levels about EDs. 2. Increase the sense of control over environmental risks. 4. Be able to implement appropriate prevention strategies. 6. Reduce anxiety related to EDs and their exposure.	50 min Students analyze the components of presented cosmetic products.
Label reading	1. Increase knowledge levels about EDs. 2. Increase the sense of control over environmental risks. 3. Be able to assess risks related to EDs in everyday life and/or professional situations. 4. Be able to implement appropriate prevention strategies. 5. Know how to identify reliable sources of information on EDs. 6. Reduce anxiety related to EDs and their exposure.	50 min Students analyze the components of presented cosmetic products.
Analyzing videos critically	2. Increase the sense of control over environmental risks. 3. Be able to assess risks related to EDs in everyday life and/or professional situations. 4. Be able to implement appropriate prevention strategies. 5. Know how to identify reliable sources of information on EDs	50 min Students critically assess videos promoting healthy products by analyzing the components of the products promoted in the videos.
Creating a podcast	2. Increase the sense of control over environmental risks. 3. Be able to assess risks related to EDs in everyday life and/or professional situations. 4. Be able to implement appropriate prevention strategies. 6. Reduce anxiety related to EDs and their exposure.	50 min Students share the changes they have made (or not) since the start of the program to limit their exposure.
Research an article and present it	1. Increase knowledge levels about EDs. 2. Increase the sense of control over environmental risks. 3. Be able to assess risks related to EDs in everyday life and/or professional situations.	50 min In this second part, students present to the rest of the group the information extracted from the articles they have selected.
Photo expression and debriefing	2. Increase the sense of control over environmental risks. 4. Be able to implement appropriate prevention strategies. 6. Reduce anxiety related to EDs and their exposure.	50 min This tool follows session 1. Students select the same photo as in session 1 and perform a self-assessment of the evolution of their beliefs and knowledge regarding endocrine disruptors.

* Posters are not a tool but can be used during the implementation of the second and third program sessions to support the intervention.

## Data Availability

Due to the nature of the data and their connection to a workplace, the data are not stored in a publicly accessible archive. Inquiries can be directed to the corresponding author.

## Appendix A. Detailed Methodology and Questionnaire for Focus Groups Informing the Development of the COPEADOSE Educational Tools

The population for the focus groups consisted of adolescents enrolled in either general or vocational education programs in secondary schools in the Loire department of France. In addition to participating in the focus groups, students completed a questionnaire where they anonymously shared their demographic information. A detailed description of the methodology used to develop the focus group questionnaire guide is provided in the subsequent paragraph.

The methodology unfolded with a meticulous multi-step approach. First, a pilot test of the questionnaire was conducted with five students, followed by a thorough review of test recordings to identify areas of confusion or hesitancy. Subsequently, the final version of the questionnaire was fine-tuned through a three-person consensus to structure the focus group scenarios effectively. Data collection was carried out through audio recordings and observational techniques.

Recruitment efforts spanned six schools, yielding participation from 275 students. Data analysis was conducted using NVivo 15 software, with one-third of the data subjected to double coding to ensure reliability. The coding comparison yielded an impressive agreement rate of 98.2%. Grounded in the theoretical framework of Kidd and Parshall (2000), the discourse unit was employed as the analytic unit, capturing both collective and individual perspectives [27]. The analysis considered sequential and non-sequential declarations from participants, providing a nuanced representation of diverse, and at times contradictory, viewpoints.

The data processing involved three main stages. First, an immersion phase was conducted by listening to audio recordings, revisiting transcripts, and paying close attention to their nuances. Second, a coding phase utilized the analytical grid proposed by Duschene and Haegel, facilitating the corpus’s comparison against initial hypotheses [28]. This iterative coding approach allowed for refinement and the addition of emergent categories. Finally, a synthesis phase consolidated the findings.

Focus Group Question Framework

○ Have you ever heard of endocrine disruptors?
○ Have you come across this term before?
  ▪ (If yes) Where and how?
  ▪ (If no or silence) Again, I want to stress that there are no right or wrong answers.
○ What words, images, or ideas immediately come to mind when we mention “endocrine disruptors”?
○ How does this topic make you feel?
○ Do you think it’s important or interesting?
○ Would you like to learn more about it?
○ If you had to define the term “endocrine disruptor”, what would you say? (Break it down into “endocrine” and “disruptor”—use writing and drawing if necessary.)
○ Do you think there are interactions between endocrine disruptors and humans?
  ▪ If yes, what kind of interactions?
○ “Endocrine” relates to hormones. Is this a word you are familiar with?
○ Who can define it or give examples?
○ What do you know about it, and what is its role?
○ If I said we were going “hunting” for endocrine disruptors, where would you go?
○ (Outdoors/at home, rare/common?)
○ How does this make you feel?
○ Does it seem like an important or interesting subject?
○ Would you want to learn more about it?
○ Do you think endocrine disruptors can be avoided, fought against, eliminated, modified, protected against, limited, reduced, or recycled?

(Complete the list with their ideas and ask, “What would you choose and why?”)

○ How would you approach this?
○ In your opinion, what are the consequences of endocrine disruptors?
  ▪ For individuals?
  ▪ On a larger scale?
○ (What impact do endocrine disruptors have on health and the environment?)
○ Do these consequences pose a risk to individuals or exposed environments?
○ Do you think you have ever been in contact with endocrine disruptors?
  ▪ If yes, in what situations?
○ Do you think you might encounter them later in your professional life?
○ Are there specific jobs you believe are more exposed to endocrine disruptors than others?
○ How do you (or would you) seek information about endocrine disruptors?
○ (Who or what would you turn to for information?)
○ For each type of media:
○ “What?” (e.g., internet = which site?)
○ “Why?” (e.g., why this site?)
○ If you were to inform and engage your peers about endocrine disruptors, how would you do it?
○ What seems most important to prioritise?
○ (What format? What information? What media—posters, videos, other?)

Presentation of an educational video realised by the Ministry of Health and available in French on Youtube

Post-Video Discussion

○ At home, is this a topic that is discussed?
○ Who talks about it: you, your parents, or another family member?
○ With whom?
○ Are any examples from the video implemented in your household?
○ Could other examples be implemented?

Concluding Roundtable

○ General question: Do you have anything else you’d like to share?
○ Have you previously been informed about health topics?
  ▪ If yes, which ones?
○ In what context?
○ With what level of interest?
○ What is your interest in addressing health or environmental topics at school? Rate it on a scale from 0 to 5.
○ What other health or environmental topics would you like to learn about? (Rank them in order of preference.)

Individual Reflection

○ What will you take away from this session?
○ Summarise in a few words.

## Appendix B. Detailed Information on Theories Used in Step 1 of the IM

Overview of theories used to target concepts highlighted by the focus groups:

These theories, including Fishbein and Ajzen’s theory of reasoned action [11], Ajzen et al.’s theory of planned behavior [12], and Bandura’s social cognitive theory [13], illuminate the determinants highlighted within the focus groups. These theories are geared towards predicting individual behaviors by considering their pre-existing attitudes, behavioral intentions, and the surrounding context [29].

By linking the identified needs from the focus groups with the theory of reasoned action, the theory of planned behavior, and Bandura’s self-efficacy theory, the behavioral determinants targeted by the COPEADOS project were underscored. This involved aspects such as attitude, knowledge, risk perception, self-efficacy, and skills, all of which were utilized to develop and assess an educational program designed to enhance adolescents’ knowledge and abilities in recognizing and protecting themselves against endocrine disruptors.

The theory of reasoned action posits that various contextual factors can influence the initiation of behavioral change. A lack of knowledge can act as a barrier to adopting new behaviors. Consequently, the project’s objective is to provide tools that facilitate the acquisition of knowledge on endocrine disruptors.

In the theory of reasoned action, attitude relates to the positive or negative evaluations concerning the actual performance of a behavior and is influenced by beliefs. These beliefs and attitudes have a significant impact on health behaviors. The COPEADOS project aims to provide tools to assess and address beliefs and attitudes regarding endocrine disruptors. However, having a positive attitude towards a behavior does not always lead to change; other factors are also involved in behavior change. This is why tools targeting other determinants were developed.

In this context, risk perception and self-efficacy are crucial for adopting new health behaviors. According to the theory of planned behavior [12], perceived control can alter not only behavioral intention but also the behavior itself. Self-efficacy pertains to people’s confidence in their ability to take action that influences events in their lives [13]. The belief in efficacy suggests that if individuals do not believe they can achieve the desired outcomes through their actions, they have little motivation to act or persist in the face of challenges. Therefore, COPEADOS’s program also aimed at targeting adolescents’ risk perception and self-efficacy towards EDs.

Finally, the social cognitive learning theory, as developed by Albert Bandura and others in 1977 [13], views behavior changes as a result of personal and environmental determinants. According to this theory, promoting change requires offering learning opportunities and cultivating skills for new behaviors and problem-solving. That is why the project ensures the capacity to act on adolescents’ skills.
